# Prevalencia de colonización vaginorrectal por Streptococcus agalactiae y su perfil de sensibilidad en mujeres embarazadas atendidas en un hospital de tercer nivel

**DOI:** 10.7705/biomedica.4514

**Published:** 2019-12-30

**Authors:** César Hernán Campo, María Fernanda Martínez, Juan Carlos Otero, Giovanna Rincón

**Affiliations:** 1 Departamento de Ginecología y Obstetricia, Hospital Universitario de Santander, Bucaramanga, Colombia Departamento de Ginecología y Obstetricia Hospital Universitario de Santander Bucaramanga Colombia; 2 Grupo de Investigación de Inmunología y Epidemiología Molecular, Universidad Industrial de Santander, Bucaramanga, Colombia Universidad Industrial de Santander Grupo de Investigación de Inmunología y Epidemiología Molecular Universidad Industrial de Santander Bucaramanga Colombia; 3 Departamento de Ginecología y Obstetricia, Escuela de Medicina, Facultad de Salud, Universidad Industrial de Santander, Bucaramanga, Colombia Universidad Industrial de Santander Escuela de Medicina Facultad de Salud Universidad Industrial de Santander Bucaramanga Colombia

**Keywords:** Streptococcus agalactiae, mujeres embarazadas, sepsis neonatal, profilaxis antibiótica, Streptococcus agalactiae, pregnant women, neonatal sepsis, prophylaxis antibiotic

## Abstract

**Introducción.:**

Streptococcus agalactiae es el principal agente etiológico causante de infección invasiva del recién nacido con cuadros clínicos que pueden cursar con septicemia, neumonía o meningitis con prevalencias hasta del 50 % a nivel mundial, donde se viene presentando un incremento en su resistencia antibiótica.

**Objetivo.:**

Estimar la prevalencia de colonización vaginorrectal por S. agalactiae y su perfil de sensibilidad, en mujeres embarazadas atendidas en un hospital de tercer nivel.

**Materiales y métodos.:**

Se muestrearon 121 mujeres gestantes mediante hisopado vaginal y rectal. Los cultivos se desarrollaron siguiendo la metodología recomendada por los Centers for Disease Control and Prevention (CDC) y se agregó el agar chromID Strepto B. Las colonias sugestivas se identificaron bioquímicamente y se determinaron los perfiles de sensibilidad según el Clinical and Laboratory Standards Institute (CLSI).

**Resultados.:**

La prevalencia de colonización por S. agalactiae en las mujeres gestantes fue del 20,66 %. Se obtuvieron 40 aislamientos del total de muestras analizadas, de los cuales, el 12,5 % no presentó sensibilidad a la penicilina. La sensibilidad a la levofloxacina, la clindamicina y la eritromicina fue de 100 %, 92,5 % y 87,5 %, respectivamente; no se encontró sensibilidad a la tetraciclina. El fenotipo iMLSB se encontró en tres y, el M, en dos de los 40 aislamientos.

**Conclusiones.:**

La prevalencia de colonización vaginorrectal por S. agalactiae en la población de estudio, fue de 20,66 %. Se obtuvieron aislamientos no sensibles a la penicilina, y con resistencia a los macrólidos y las lincosamidas mediante el método de Kirby-Bauer. Por ello, es importante la búsqueda activa en las mujeres gestantes colonizadas por estreptococos del grupo B y la vigilancia epidemiológica constante para detectar cambios en los perfiles de sensibilidad de los aislamientos.

## Abstract (en)

**Introduction:**

Streptococcus agalactiae is the main etiological agent causing invasive infection of the newborn with symptoms that may be associated with septicemia, pneumonia or meningitis and prevalences up to 50% worldwide where there is an increase in antibiotic resistance.

**Objective:**

To estimate the prevalence of vagino-rectal colonization by S. agalactiae and its sensitivity profile in pregnant women attending a third-level hospital.

**Materials and methods:**

One hundred and twenty one pregnant women were sampled by vaginal and rectal swabs. The cultures were carried out following the methodology recommended by the CDC, and chromID Strepto B agar was added. The suggestive colonies were identified biochemically and the sensitivity profiles according to CLSI were determined. As control, S. pneumoniae ATCC 49619 and S. agalactiae ATCC 12403 were used.

**Results:**

Pregnant colonization prevalence by S. agalactiae was 20.66%, with a total of 40 isolates of which 12.5% were non-sensitivity to penicillin. Sensitivity to levofloxacin, clindamycin and erythromycin was 100%, 92.5% y 87.5%, respectively, with the phenotypes iMLSB (3/40) and M (2/40). No sensitivity to tetracycline was found.

**Conclusions:**

The prevalence of vagino-rectal colonization by S. agalactiae in the study population was 20.66%, obtaining isolates not sensitive to penicillin and resistance to macrolides and lincosamidas by the Kirby-Bauer technique, so the importance of carrying out active active in pregnant women colonized by Streptococcus agalactiae and to perform constant epidemiological surveillance to detect changes in the sensitivity profiles of the isolates.

Streptococcus agalactiae o estreptococo del grupo B coloniza habitualmente las vías genitourinarias y gastrointestinales del humano. Esta bacteria se considera de gran importancia por ser el principal agente etiológico de la infección invasiva del recién nacido ^1^^-^^4^, pues se adquiere en el momento del nacimiento a través del canal vaginal.

Generalmente, la colonización materna es asintomática y muestra prevalencias variables, entre el 5 y el 30 %, a nivel mundial ^4^^,^^5^. Las mujeres gestantes colonizadas pueden transmitir esta bacteria a sus hijos, lo cual favorece el desarrollo de infección neonatal temprana en 1 a 2 % de los neonatos ^3^^-^^7^.

En los Estados Unidos, se ha descrito que la infección por *S. agalactiae* es la principal causa de mortalidad y morbilidad neonatal. En la década de 1970, alcanzó una tasa de letalidad de 50 %, aproximadamente, la cual ha disminuido a partir del año 1990 hasta el 5 %, gracias a la implementación de las medidas de prevención por parte del *American College of Obstetricians and Gynecologists* (ACOG) y los *Centers for Disease Control and Prevention* (CDC) ^4^.

Estas medidas están basadas en la tamización mediante cultivos vaginorrectales en mujeres embarazadas con 35 a 37 semanas de gestación y en la profilaxis antibiótica intraparto ^2^^,^^4^^,^^8^. Los CDC sugieren el uso de medios de cultivo selectivos y específicos, para aumentar significativamente las tasas de detección de *S. agalactiae*^8^; para la profilaxis, recomiendan administrar penicilina G y, en las mujeres gestantes alérgicas a los betalactámicos, se debe usar clindamicina como antibiótico de segunda línea ^2^.

En Colombia, son pocos los estudios de prevalencia de colonización por estreptococo del grupo B; se han encontrado tasas variables: de 5,7 %, 8,6 % y 17,6 % en Medellín ^1^^,^^2^^,^^7^, de 0,38 %, 16,4 % y 15,2 % en Bogotá ^3^^,^^5^^,^^9^, de 36,6 % en Cartagena ^10^, y de 3,9 % en Cali ^11^. En el departamento de Santander, no se encontraron reportes sobre la prevalencia de colonización y los perfiles de sensibilidad de este microorganismo en mujeres gestantes, y es importante la realización de estudios en la región, no obstante, en el futuro se espera tener tasas de prevalencia globales de colonización en Colombia, al encontrarse la tamización de búsqueda de *S. agalactiae*, dentro de los lineamientos de obligatorio cumplimiento de la ruta materno-perinatal y en la Ruta Integral de Atención en Salud (RIAS). 

La tamización y la vigilancia de los perfiles de sensibilidad a los antibióticos son cruciales para una prevención adecuada en las mujeres con alto riesgo de anafilaxis y, especialmente, por el aumento de cepas no sensibles a la penicilina, la cual se usa empíricamente. La falta de atención médica y el uso abusivo de estos fármacos, han incrementado el número de aislamientos resistentes que reducen las alternativas de profilaxis ^2^. 

Considerando lo anterior y teniendo en cuenta la falta de datos en Santander, el objetivo de este estudio fue estimar la prevalencia de colonización vaginorrectal de *S. agalactiae* y establecer su perfil de sensibilidad antimicrobiana en mujeres embarazadas atendidas en un hospital de tercer nivel.

## Materiales y métodos

### Tipo de estudio

El estudio es de tipo observacional, descriptivo y de corte transversal.

### Población y muestra

Se muestrearon 121 mujeres con 35 a 37 semanas de gestación, atendidas en los servicios de consulta externa o urgencias de un hospital de tercer nivel. El 68,5 % de las pacientes provenía de Bucaramanga y de otros municipios del área metropolitana, y la edad promedio era de 25,09 años, con una mediana de 23 años. Se incluyeron pacientes adultas y menores de edad que cumplían con los criterios de inclusión, previa firma del consentimiento informado.

Como criterio de exclusión, se tuvieron en cuenta: ruptura prematura de membranas; infección bacteriana vaginal o rectal; infección urinaria o bacteriuria asintomática diagnosticada en los ocho días anteriores a la toma de la muestra, administración de antibiótico en el mes previo a su valoración y tratamiento local tópico con óvulos o cremas vaginales al momento de tomar la muestra.

### Aislamiento e identificación

Se tomó un hisopado del introito vaginal y uno de la región anorrectal, los cuales fueron transportados en medio Amies Portagerm™ (bioMerieux, France). Las muestras se cultivaron directamente en caldo selectivo de ToddHewitt (bioMerieux, France), con suplemento de los antibióticos gentamicina (8 µg/ml) y ácido nalidíxico (15 µg/ml). Se incubaron durante 18 a 24 horas a 37±2 ºC en condiciones de aerobiosis.

Posteriormente, se cultivaron en medio cromogénico ChromID Strepto B™ (bioMerieux, France) y en agar sangre de cordero al 5 % (Agar Columbia, bioMerieux, France). Las placas se incubaron de 18 a 24 horas, las de agar sangre, en atmósfera con 5 % de CO^2^ y, aquellas con medio cromogénico ChromID Strepto B™ en atmósfera aerobia y protegidas de la luz.

Se consideraron colonias indicativas de S. agalactiae las de color rosa pálido o rojo en el medio cromogénico, colonias medianas o pequeñas β-hemolíticas en agar sangre o ambas. Se identificaron mediante tinción de Gram, prueba de catalasa, prueba de CAMP con *Staphylococcus aureus* ATCC 25923, y mediante el panel de pruebas bioquímicas automatizadas BD Phoenix™ (Becton Dickinson, USA).

### Cepas control

Se utilizaron las cepas de *S. agalactiae* ATCC 12403 para el control de calidad del medio cromogénico y del agar sangre, y *S. pneumoniae* ATCC 49619, como control de los antibiogramas por el método de Kirby-Bauer.

### Sensibilidad antimicrobiana

El antibiograma se obtuvo mediante el método de difusión en disco en medio de Mueller-Hinton con 5 % de sangre, y se emplearon sensidiscos con 10 U de penicilina G, 15 µg de eritromicina, 2 µg de clindamicina, 30 µg de tetraciclina y 5 µg de levofloxacina (Oxoid, UK), siguiendo las normas establecidas por el *Clinical Laboratory Standards Institute* (CLSI, 2016). Se practicó el test D usando discos de 15 µg de eritromicina y 2 µg de clindamicina, a una distancia de 12 mm uno del otro, siguiendo la guía del CLSI para determinar los fenotipos de resistencia a los antibióticos MLS_B_.

### Análisis estadístico

La base de datos se generó en Microsoft Office Excel 2013 y se analizó en Stata 12.1™. Se hizo un análisis univariado para la descripción de las características sociodemográficas, los antecedentes ginecoobstétricos, la gestación actual y los resultados de laboratorio clínico, con intervalos de confianza del 95 %.

### Consideraciones éticas

Este estudio fue evaluado y aprobado por el Comité de Ética en Investigación Científica de la Universidad Industrial de Santander (CEINCIUIS) y del Hospital Universitario de Santander. Esta investigación fue considerada de riesgo mínimo para las pacientes, según lo establecido en el artículo 11 de la Resolución 008430 de 1993 del Ministerio de Salud. 

## Resultados

De las 121 mujeres muestreadas, en 25 mujeres gestantes se obtuvo, al menos, un cultivo positivo para *S. agalactiae*, lo que corresponde a una prevalencia global de 20,66 %. Del total de mujeres gestantes con cultivos positivos, 19 (15,7 %) eran portadoras de *S. agalactiae* en la vagina, 21 (17,36 %), en la zona anorrectal y, 15 (12,4%), tanto en la región anorrectal como la vaginal. Se obtuvieron 40 aislamientos de *S. agalactiae*, entre los cuales se encontró una cepa con fenotipo no hemolítico, la cual se detectó en el agar cromogénico y se identificó posteriormente como *S. agalactiae*.

El análisis de las variables sociodemográficas de las pacientes colonizadas por *S. agalactiae*, no mostró diferencias estadísticamente significativas en comparación con la población no colonizada. En este estudio, no se observó una relación significativa entre las pacientes con antecedentes ginecoobstétricos de riesgo de colonización y las pacientes colonizadas, lo cual concuerda con la guía de los CDC, donde refieren que la mayoría de las mujeres colonizadas no tienen factores de riesgo. Las características de las mujeres gestantes a quienes se les practicó el cultivo, se resumen en el cuadro 1.

**Cuadro 1:** Características de las pacientes con muestras positivas o negativas para colonización por *Streptococcus agalactiae*

	Positivas		Negativas		
Característica	(n=25)		(n=96)		P
	n %			n %	
			
Edad (años)	Media: 25,1;		Media: 25;		0,9576
	IC95% (21,5-28,7)		IC95% (23,7-26,5).		
Procedencia					
Bucaramanga	9	(36)	41	(42,7)	
Floridablanca	4	(16)	10	(10,4)	
Girón	3	(12)	9	(9,4)	
Piedecuesta	2	(8)	5	(5,2)	
Otros	7	(28)	31	(31,3)	0,680
Estado civil					
Soltera	2	(8)	15	(15,6)	
Casada	3	(12)	15	(15,6)	
Unión libre	20	(80)	66	(68,8)	0,269
Paridad					
Primigestante	8	(32)	29	(30,2)	
Multigestante	17	(68)	67	(69,8)	0,863
Estrato bajo	20	(80,0)	89	(92,7)	0,058
Antecedente de RPM	0	(0)	25	(100,0)	0,164
Antecedente de parto					
prematuro	1	(4)	12	(12,5)	0,222
Antecedente de enfermedad					
de transmisión sexual	1	(4)	11	(11,5)	0,266
Antecedente de infección					
neonatal temprana	0	(0)	2	(2,1)	0,467
Edad gestacional					
Semana 35-35 6/7	7	(28)	37	(38,5)	
Semana 36-36 6/7	5	(20)	17	(17,7)	
Semana 37-37 6/7	13	(52)	42	(43,8)	0,461
Buen control prenatal	3	(12)	24	(25,5)	0,151

RPM; ruptura prematura de membranas

**Figura 1.**
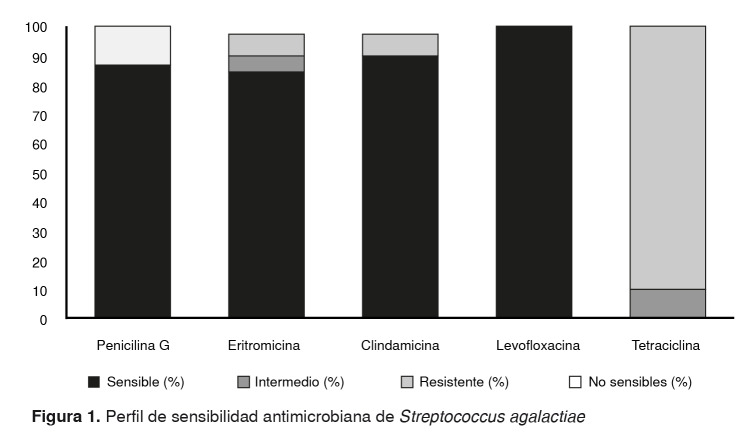
Perfil de sensibilidad antimicrobiana de Streptococcus agalactiae

Frente a los resultados obtenidos según el perfil de sensibilidad, se encontró que el 87,5 % fueron sensibles a la penicilina, mientras que el 12,5 % no lo fueron. La sensibilidad a la levofloxacina, la clindamicina y la eritromicina fue de 100 %, 92,5 % y 87,5 %, respectivamente (figura 1).

El fenotipo iMLSB se expresó en tres aislamientos y el M en dos (cuadro 2). No se encontró sensibilidad a la tetraciclina en las cepas estudiadas.

**Cuadro 2:** Fenotipos de Streptococcus agalactiae con resistencia a los antibióticos de tipo macrólidos, lincosamidas y estreptograminas B

Fenotipo	Interpretación		n (%)	Mecanismo de resistencia
			
ERI	CLI		
		
cMLS_B_	R	R	0	Metilasas por ARN ribosómico
iMLS_B_	R	S*	3 (7,5)	Metilasa inducibles por ARN ribosómico
M	R	S	2 (5)	Bombas de expulsión
L	S	R	0	Inactivación del antibiótico

cMLS B: fenotipo constitutivo; iMLS B: fenotipo inducible; ERI: eritromicina; CLI: clindamicina; R: resistente; S: sensible; *: sensible con achatamiento del halo

## Discusión

Streptococcus agalactiae puede producir infección intrauterina o infección neonatal de instalación temprana o tardía. La infección se adquiere de manera vertical por contacto del feto con secreciones vaginales en el momento del parto o por ascenso de la bacteria a través de las membranas ovulares rotas ^4^^,^^12^^,^^13^. Se hace importante detectar mujeres portadoras mediante el cultivo vaginorrectal entre las semanas 35 y 37 de gestación, ya que permite una aproximación más real al estado de colonización de la mujer en el momento del parto ^4^^,^^5^.

Se reporta que el cultivo sistemático de tamización ofrece mayor efectividad en la prevención de la transmisión vertical, en comparación con la prevención basada solo en factores de riesgo, como el embarazo de menos de 37 semanas, la ruptura prematura de membranas de más de 18 horas y la fiebre por encima de los 38 ºC ^1^^,^^4^. En el estudio de Scharg, *et al*. (2002), se encontró que hasta el 18 % de las mujeres gestantes portadoras no presentaban factores de riesgo. Por esta razón, esta metodología permitiría la identificación precoz en estas pacientes ^14^.

Para la tamización, los CDC recomiendan el cultivo en caldo de Todd-Hewitt con suplemento de antibióticos, y sugiere el uso de medios cromogénicos para aumentar la detección de portadoras de *S. agalactiae*^4^. 

Siguiendo esta metodología, se estimó una prevalencia de colonización del 20,66 % en este estudio, similar a la encontrada en varios países de Europa y en los Estados Unidos ^15^^-^^18^. En países como Argentina, Perú, Uruguay, Venezuela, Brasil, México y Chile, se han reportado prevalencias que oscilan entre 1,4 % y 62,7 % ^19^^-^^29^. 

La mayor prevalencia hallada en este trabajo, en comparación con otros reportes del país ^1^^-^^3^^,^^5^^,^^7^^,^^9^^-^^11^, puede explicarse por el sitio de toma de la muestra (rectal, vaginal o ambas), la población estudiada y el uso de medios de cultivos específicos, ya que se ha reportado que un escaso porcentaje de *S. agalactiae* (menor del 2 %) no produce betahemólisis ^30^. Además, al usar solo medios convencionales, se pasarían por alto estos aislamientos que pueden ser de difícil identificación, como se confirmó en este estudio al encontrar *S. agalactiae* de un fenotipo no hemolítico y que solo pudo ser detectado con el medio cromogénico.

Asimismo, el cultivo fue positivo para *S. agalactiae* en la muestra anorrectal del 17,36 % de las mujeres gestantes y, a su vez, en 71 % de ellas, el cultivo de la muestra vaginal fue positivo; por lo tanto, se hace importante hacer cultivos de ambos sitios para disminuir el riesgo de transmisión con una profilaxis oportuna ^9^.

En el presente estudio, no se hizo seguimiento a los recién nacidos de madres colonizadas, para determinar el grado de transmisión y colonización neonatal. 

Del total de mujeres gestantes con el antecedente de parto prematuro, solamente en una (4 %) el cultivo fue positivo para *S. agalactiae* (cuadro 1). 

Se ha reportado que entre el 40 y el 70 % de las mujeres gestantes colonizadas por *S. agalactiae* transmiten el microorganismo a sus hijos durante el parto y que 1 a 2 % de los recién nacidos colonizados desarrollan la infección neonatal ^4^^,^^6^^,^^7^. Es así que los CDC recomiendan que toda mujer con el antecedente de un hijo con diagnóstico de enfermedad invasiva por *S. agalactiae*, reciba profilaxis ^4^.

La profilaxis intraparto se ha convertido en una estrategia eficaz que ha logrado disminuir la infección neonatal temprana por *S. agalactiae*. Cabe destacar que el 12,5 % las cepas encontradas no fueron sensibles a la penicilina (figura 1); debe tenerse en cuenta que los estreptococos siempre se han considerado sensibles a los antibióticos betalactámicos ^31^ y que, hasta el momento, no se han establecido criterios de resistencia de este microorganismo contra estos fármacos por parte del CLSI.

La presencia de aislamientos fenotípicamente no sensibles a la penicilina, crea la necesidad de llevar a cabo estudios de vigilancia epidemiológica, de caracterizar los cinco aislamientos encontrados en este trabajo, determinar el serogrupo y la concentración inhibitoria mínima (CIM) a la penicilina, y practicar las pruebas moleculares para dilucidar la razón del perfil encontrado.

Se ha informado *S. agalactiae* con tolerancia o sensibilidad disminuida a la penicilina desde 1994 ^32^. En un estudio en Japón, se identificaron cepas de aislamientos clínicos de *S. agalactiae* con una CIM por encima de los criterios establecidos por el CLSI (≤0,12 µg/ml) ^33^, lo cual demostró que la reducción de la sensibilidad se debía a la sustitución de aminoácidos en la proteína fijadora de penicilina, PBP2X ^33^; y, en otro estudio más reciente en el noroeste de Etiopía, se encontró una sensibilidad de 89,6 % a este antimicrobiano ^34^. Sin embargo, aún predominan las cepas sensibles y, por ello, no se hace necesario verificar de forma rutinaria la sensibilidad a este antibiótico.

Por otra parte, se han reportado algunas mujeres gestantes que pueden presentar procesos alérgicos a la penicilina, por lo cual se recomienda la clindamicina como tratamiento hasta finalizar el parto ^4^. Entre los aislamientos analizados en este estudio, 3 (7,5 %) cepas presentaron sensibilidad *in vitro* a la clindamicina y fueron positivas con el test D, por lo cual se reportaron como resistentes y su fenotipo fue iMLS_B_.

La capacidad de estas cepas para adquirir este tipo de resistencia radica en la presencia de metilasas, enzimas que están codificadas por los genes *erm* (*Erythromycin Ribosome Methylase*), cuya producción se ve favorecida por inductores fuertes, como la eritromicina, o por inductores débiles, como la clindamicina ^35^^,^^36^. 

Teniendo en cuenta que la clindamicina se considera un inductor débil a largo plazo, se hace muy importante detectar este mecanismo de resistencia en estos aislamientos, antes de administrarla, pues podría tener como consecuencia la falla terapéutica ^37^. 

En este trabajo se encontró una prevalencia de colonización vaginorrectal por *S. agalactiae* del 20,66 %, lo cual evidencia la importancia de su búsqueda activa en mujeres embarazadas para establecer la profilaxis durante la gestación. Asimismo, se resalta la importancia de la utilización de medios cromogénicos, ya que permite detectar cepas *S. agalactiae* con fenotipo no hemolítico. 

Las cepas aisladas presentaron fenotipo con sensibilidad a la penicilina y resistencia a los macrólidos y lincosamidas. Esto demuestra la necesidad de la vigilancia epidemiológica, pues estos antibióticos se emplean como tratamiento en la profilaxis intraparto y, a su vez, la necesidad de caracterizar las cepas aisladas en este estudio.
